# When Teams Fail to Self-Regulate: Predictors and Outcomes of Team Procrastination Among Debating Teams

**DOI:** 10.3389/fpsyg.2018.00464

**Published:** 2018-04-05

**Authors:** Edwin A. J. Van Hooft, Heleen Van Mierlo

**Affiliations:** ^1^Work and Organizational Psychology, Department of Psychology, University of Amsterdam, Amsterdam, Netherlands; ^2^Institute of Psychology, Erasmus University Rotterdam, Rotterdam, Netherlands

**Keywords:** team regulation, procrastination, team motivation, goal orientation, team performance, stress, multilevel model of team procrastination

## Abstract

Models of team development have indicated that teams typically engage in task delay during the first stages of the team’s life cycle. An important question is to what extent this equally applies to all teams, or whether there is variation across teams in the amount of task delay. The present study introduces the concept of team procrastination as a lens through which we can examine whether teams collectively engage in unplanned, voluntary, and irrational delay of team tasks. Based on theory and research on self-regulation, team processes, and team motivation we developed a conceptual multilevel model of predictors and outcomes of team procrastination. In a sample of 209 student debating teams, we investigated whether and why teams engage in collective procrastination as a team, and what consequences team procrastination has in terms of team member well-being and team performance. The results supported the existence of team procrastination as a team-level construct that has some stability over time. The teams’ composition in terms of individual-level trait procrastination, as well as the teams’ motivational states (i.e., team learning goal orientation, team performance-approach goal orientation in interaction with team efficacy) predicted team procrastination. Team procrastination related positively to team members’ stress levels, especially for those low on trait procrastination. Furthermore, team procrastination had an indirect negative relationship with team performance, through teams’ collective stress levels. These findings add to the theoretical understanding of self-regulatory processes of teams, and highlight the practical importance of paying attention to team-level states and processes such as team goal orientation and team procrastination.

## Introduction

Procrastination is a widespread phenomenon, occurring regularly at school, at work, and in our daily lives (see Ferrari et al., 1995; Van Eerde, 2000; Steel, 2007). About 10–20% of the general population is estimated to suffer from habitual procrastination (Harriott and Ferrari, 1996; Ferrari et al., 2007), whereas almost everyone procrastinates on tasks every now and then (i.e., 95% in Ferrari et al., 1995; Van Hooft, 2010). Procrastination refers to a failure in self-regulation, defined as the voluntary delay of an intended course of action despite the negative consequences of the delay (Steel, 2007). Such negative consequences of procrastination relate to missing deadlines (Ferrari, 1993; Van Eerde, 2003), poor performance (Steel, 2007), reduced career success (Nguyen et al., 2013), and decreased mental health (Tice and Baumeister, 1997; Sirois et al., 2003; Sirois, 2014).

Previous research on procrastination has exclusively studied the phenomenon at the individual level. However, people oftentimes do not operate individually; both in educational settings and in work settings, collaboration in small groups or teams on tasks and projects is ubiquitous (e.g., Hansen, 2006; Kozlowski and Ilgen, 2006; Mathieu et al., 2008). Teams or small groups are usually characterized as distinguishable collectives, composed of two or more individuals who interact and work interdependently toward a common and valued goal (Salas et al., 1992; Ilgen, 1999). Models of team development have noted that teams may engage in task delay or avoidance (Gersick, 1988, 1989; Chang et al., 2003). For example, Gersick’s punctuated equilibrium model states that project teams during the first half of the allotted time display a period of inertial movement during which they delay revising their initial plans. Data showed that attention to time and pacing in teams generally is low during the early phases of a project and increases curvilinearly (Steel and König, 2006). In the present study, we extend these models by proposing that teams may differ in the extent to which they engage in delay of goal-directed team activities, and introduce the concept of team procrastination. Given the prominence of working in teams and team-based learning, and given the prevalence and negative consequences of procrastination at the individual level, it is important to investigate the extent to which procrastination occurs in teams, and examine the predictors and outcomes of team-level procrastination.

Theoretically, this study aims to extend our understanding of self-regulation of teams by initiating theory building on team-level procrastination and its antecedents and outcomes. In building our theoretical framework, we draw upon team effectiveness models (i.e., Input-Process-Output [IPO] and Input-Mediators-Output-Input [IMOI] models; Ilgen et al., 2005; Mathieu et al., 2008) and team motivation models (i.e., Chen and Kanfer, 2006; Chen and Gogus, 2008). Specifically, we empirically investigate the existence of team procrastination, and examine its predictors and outcomes in a multi-wave study among small interdependent student teams, who collaborated on two debating tasks (i.e., a written assignment and an oral debate) with assigned deadlines. Practically, this study may contribute to team effectiveness and team member well-being by yielding guidelines on how to decrease procrastination of teams.

## Team Procrastination

Based on Steel’s (2007) behavioral definition of individual-level procrastination, we define *team procrastination* as the unplanned, voluntary, and irrational delay of intended goal-directed team activities. Team procrastinatory behavior thus refers to collective engagement of the team in task delay, despite the team’s intentions to work on the task, and despite the team expecting to be worse off for the delay. For example, a student team may set a meeting to jointly work on an assignment, but finds itself chatting about issues unrelated to the task, or a project team plans to distribute tasks between the team members but postpones this activity each time they get together.

We explicitly define team procrastination as a team-level construct referring to collective behavior of the team. As such, it differs from individual-level (trait) procrastination, which refers to a person’s individual tendency or behavior, and from social loafing (Karau and Williams, 1993) which refers to individuals (rather than teams) intentionally expending less effort (rather than unintentionally delaying a task) in a team setting. Rather, team procrastination reflects a team-level construct referring to failing self-regulation of teams. In terms of theorizing on types of self-regulatory failure (Baumeister and Heatherton, 1996; Inzlicht et al., 2014), team procrastination can be understood as a lack of regulatory capacity or a lack of motivational priority of the team to change or modulate the team’s current state/behavior toward the team goals. In line with previous theorizing on team motivation (Chen and Kanfer, 2006; Chen and Gogus, 2008), team procrastination refers to the goal-striving system, and is proposed to be an indicator of team regulatory failure or counterproductive team behavior during goal striving. In other words, team procrastination refers to failing self-regulation *of* teams rather than failing self-regulation *in* teams. That is, while self-regulation *in* teams refers to individual-level self-regulation in a team context, self-regulation *of* teams refers to team-level regulation of goal-directed activities over time and across changing circumstances, implying team-level modulation of attention, thoughts, affect, and behavior in order to attain the team goals. Lastly, in terms of IPO and IMOI models (e.g., Ilgen et al., 2005; Mathieu et al., 2008) team procrastination reflects a team process or behavioral mediational process, involving team members’ interactions directed toward task accomplishment. More specifically, in terms of Marks et al. (2001) taxonomy of team processes, team procrastination reflects a team process construct indicating a failure to translate team goals and plans that are developed through transitional processes into actual actions (i.e., taskwork).

**FIGURE 1:**
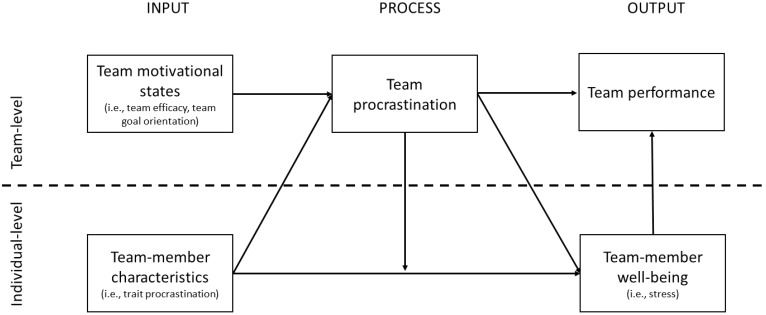
Conceptual multilevel model of predictors and outcomes of team procrastination.

Although not focusing on team procrastination, previous research did examine individual procrastination in social contexts. For example, based on scenario studies, Ferrari (1992) and Ferrari and Patel (2004) found that procrastinators negatively evaluate other people’s procrastinating behavior and that they would allocate fewer resources to a procrastinating peer. Importantly, this research suggests that procrastination does occur in social contexts. In the present study we build on these notions and previous models on team development, team motivation, and team effectiveness by examining if teams collectively engage in procrastination as a group, and what the predictors and outcomes are of such collective behavior. Specifically, as a first goal we examined to what extent team procrastination exists as a team-level motivational phenomenon. In order to do so, we investigated whether team members have a shared conception of the team’s collective procrastinatory behavior when working on an interdependent task, and whether this shared conception demonstrates some stability over time. Similar to other shared team properties, emergent states, and team processes (e.g., Klein and Kozlowski, 2000; Marks et al., 2001), we expect that teams develop a shared conception of team procrastination (ranging from low to high levels of team procrastination) when working on team tasks with a set deadline. Such a shared conception of team procrastination may emerge through processes such as team norm-setting, team-member socialization, and interaction among team members. As a second goal of the present study, we examined predictors and outcomes of team procrastination. In the following we will develop the rationale for our hypotheses and conceptual multilevel model as displayed in **Figure 1**.

## Predictors of Team Procrastination

Based on IPO, IMOI, and team motivation models (e.g., Chen and Kanfer, 2006; Mathieu et al., 2008), we distinguish between characteristics at the individual team-member level and at the team level, and propose that team procrastination is predicted by both individual-level characteristics of the team members and team-level motivational states.

Regarding the individual-level characteristics, team motivation models (e.g., Chen and Kanfer, 2006) suggest that team motivation processes depend on individual characteristics such as motivational traits. Likewise we propose that team regulation processes such as team procrastination depend on individual team members’ self-regulatory traits. As an index of trait self-regulation, we propose that team member’s level of trait procrastination is the most proximal individual-level trait predictor of team procrastination. Trait procrastination is an individual disposition that refers to an individual’s tendency to postpone that what is necessary to reach some goal (Lay, 1986), and as such refers to low trait self-regulation (Steel, 2007). Although having a disposition toward procrastination does not necessarily mean that people engage in procrastination on every task in all situations, in general we propose the team’s composition in terms of trait procrastination to positively predict team procrastination. That is, teams that are composed of team members who have a stronger disposition to engage in procrastination, more likely develop team norms, socialization processes, and communication patters that promote the likelihood that the team will irrationally delay intended goal-directed team activities.

*Hypothesis 1:* Team composition in terms of individual team members’ average trait procrastination is positively related to team procrastination.

Team motivation models (Chen and Kanfer, 2006) suggest that goal-striving processes in teams are importantly affected by proximal motivational states such as the team’s task efficacy and goal orientation. Such motivational states refer to beliefs or attitudes regarding experiences within a task environment and perceived capacity to perform tasks within the task environment (Chen and Gogus, 2008). Based on these models we propose that, in addition to team members’ trait procrastination, team procrastination is predicted by the team’s shared perceptions and framing of the task, reflecting emergent team motivational states.

As a first important motivational predictor of team procrastination, we examine the role of the team’s collective efficacy, referring to shared perceptions of task-specific team capability (Gully et al., 2002). Tasks that are perceived as complex and too challenging for one’s level of ability, are likely to be experienced as threatening, leading to task avoidance, slackening effort, and procrastination (Bandura, 1982; Van Eerde, 2000). Similarly, temporal motivation theory (Steel and König, 2006) suggests that activities with a low expectancy (i.e., as a result of feeling not very efficacious on a task) have a low utility, and are therefore more likely to be procrastinated (Steel, 2007). We therefore propose that, compared to more efficacious teams, less efficacious teams will be more likely to perceive the task as aversive, leading to more avoidance behaviors such as team procrastination.

*Hypothesis 2:* Team efficacy is negatively related to team procrastination.

Team goal orientation is a second potential motivational predictor of team procrastination. Goal orientation refers to “dispositional or situational goal preferences in achievement situations” (Payne et al., 2007, p. 128). Our focus is on goal orientation as a team-level motivational state, defined as the team’s goal preferences for a specific achievement situation. Thus, team goal orientation is conceptualized as a climate-like, collective, team-level construct that represents the state goal orientation of the team as a whole (cf. Bunderson and Sutcliffe, 2003; Mehta et al., 2009; Porter et al., 2010; Van Mierlo and Van Hooft, 2015). Consistent with recent research on team goal orientation (e.g., Van Mierlo and Van Hooft, 2015; Maltarich et al., 2016; see also Elliot and McGregor, 2001), we use a 2 × 2 framework of team goal orientation to develop our hypotheses. This framework distinguishes between four types of team goal orientations: (a) team learning-approach (i.e., a team focus on increasing competence and mastering something new), (b) team learning-avoidance (i.e., a team focus on avoiding incompetence or loss of competence), (c) team performance-approach (i.e., a team focus on demonstrating competence and thereby gaining positive judgments, and (d) team performance-avoidance (i.e., a team focus on avoiding demonstration of incompetence and negative judgments). Although distinct, the four goal orientations are conceptually related as they are based on two underlying dimensions (i.e., learning-performance and approach-avoidance; Elliot and McGregor, 2001), suggesting that teams can score high or low on several goal orientations. The 2 × 2 framework extends previous trichotomous conceptualizations of goal orientation (Elliot and Harackiewicz, 1996; Vandewalle, 1997) by introducing the learning-avoidance goal orientation. Importantly, this orientation is not about avoiding to learn but about avoiding incompetence and avoiding to not have learned all there is to learn. Previous research at both the team and individual level has indicated that learning-avoidance goal orientation is a relevant and prevalent dimension (i.e., 31.2 and 33.6% in two studies by Van Yperen (2006); see also Van Mierlo and Van Hooft, 2015; Maltarich et al., 2016).

Based on the conceptualization of team goal orientation as an emergent motivational state, the four team goal orientations are posited to differentially affect the use of self-regulatory strategies during goal striving (cf. Chen and Kanfer, 2006). This occurs because team goal orientation creates a shared framework that guides the interpretation of events in achievement situations, and that directs the quality and intensity of subsequent team behavior (cf. Dweck, 1986; Dweck and Leggett, 1988; Button et al., 1996; Vandewalle, 1997; Elliot and McGregor, 2001). Based on goal orientation theory and the team motivation and regulation literature, we will argue that team learning-approach negatively and both avoidance goal orientations positively predict team procrastination, but that the relationship of performance-approach goal orientation is motivationally more complex.

Teams with a *learning-approach goal orientation* likely display adaptive mastery-oriented response patterns during goal striving. Because of their shared focus on developing competence through acquiring new skills and mastering new situations, they likely create a climate of interest and enjoyment when learning new tasks, display collective effort and persistence toward the team goals, and view feedback as diagnostic information that further guides their team efforts. Such teams are therefore unlikely to engage in escapist behaviors and avoidant coping styles such as collective procrastination on the team tasks. Previous team research has reported positive links between team learning-approach orientation and motivational and self-regulatory outcomes such as team planning, team effort, team reflexivity, and team strategizing (DeShon et al., 2004; Mehta et al., 2009; Van Mierlo and Van Hooft, 2015). Because a team learning-approach goal orientation stimulates adaptive self-regulatory processes of the team, we expect that it relates negatively to collective procrastinatory behavior. In other words, teams that view the task as a learning opportunity that may increase their competencies will likely engage in the task and adaptively regulate the team effort, rather than irrationally delay working on the task.

*Hypothesis 3:* Team learning-approach goal orientation is negatively related to team procrastination.

Based on theorizing on approach and avoidance motivation (e.g., Vandewalle, 1997; Elliot, 1999; Elliot and McGregor, 2001), the team avoidance goal orientations in the 2 × 2 framework may be expected to induce procrastination. That is, procrastination typically represents an avoidant coping style to handle difficult or otherwise aversive tasks and situations (Van Eerde, 2000). Teams with a collective *learning-avoidance goal orientation* are focused on avoiding incompetence on the team tasks and aim to prevent failing to learn all they need to learn to master the team tasks. Such teams likely worry that they may not reach their potential, resulting in a preoccupation with risk and error prevention, damage control, and detailed weighing of pros and cons before actions are taken (Van Mierlo and Van Hooft, 2015). These team states and processes will likely induce decisional delays and postponement of actual taskwork rather than collective action toward the team goals. We therefore propose:

*Hypothesis 4:* Team learning-avoidance goal orientation is positively related to team procrastination.

Team *performance-avoidance orientation* is also expected to relate positively to team procrastination. Previous theorizing has suggested that a collective performance-avoidance orientation is maladaptive for motivational and self-regulatory team states and processes. For example, it leads teams to focus more on avoiding negative outcomes at the cost of an adaptive task focus and team effort, and it induces an overemphasis on preventing failures and defensive behaviors to preserve the image of being a competent team (Dragoni, 2005; Mehta et al., 2009; Van Mierlo and Van Hooft, 2015). Similarly, goal orientation theory (e.g., Dweck, 1986; Dweck and Leggett, 1988) poses that a performance goal orientation leads to normative task evaluations (i.e., evaluating task difficulty and performance relative to other teams), making avoidant behavior and withdrawal more likely, particularly for effortful, evaluative, and failure-prone tasks. These adverse effects are especially likely for a team performance-avoidance orientation, focused on avoiding the demonstration of incompetence and negative judgments. Such an orientation leads teams to see achievement situations as a threat, which induces anxiety, obtrusive thoughts, reduced task interest, disorganization, and self-handicapping (cf., Rawsthorne and Elliot, 1999; Elliot and McGregor, 2001; Urdan, 2004; Payne et al., 2007; Van Dyck et al., 2010), all aspects that may induce procrastination (cf. Steel, 2007). Thus:

*Hypothesis 5:* Team performance-avoidance goal orientation is positively related to team procrastination.

In contrast to the other three goal orientations, the motivational and self-regulatory processes induced by *performance-approach goal orientation* are generally more complex. Extant theorizing has described performance-approach goal orientation as motivationally hybrid or incongruent (e.g., Elliot, 1999; Harackiewicz et al., 2002), because it is undergirded by both adaptive cognitions such as need for achievement and maladaptive cognitions such as fear of failure (Elliot and McGregor, 2001). On the one hand, a performance-approach goal orientation may motivate teams to achieve, as they want to demonstrate their competence and gain positive evaluations. On the other hand, when failure is likely, evaluative anxiety may arise, leading to self-protective withdrawal cognitions and behaviors. Several scholars (e.g., Elliot and Harackiewicz, 1996; Rawsthorne and Elliot, 1999) have therefore posed that the effects of performance-approach goals are contingent on personal and situational characteristics.

In line with this notion, original goal orientation theory (Dweck, 1986; Dweck and Leggett, 1988) suggested that a performance goal orientation may lead to adaptive mastery-oriented response patterns when perceived ability is high (i.e., low likelihood of failure), and to maladaptive helplessness response patterns when perceived ability is low (i.e., high likelihood of failure). Based on this rationale, we propose that whether team performance-approach goal orientation leads to team procrastination may depend on perceived task ability. Extending goal orientation theory (Dweck, 1986; Dweck and Leggett, 1988) to the team level, we expect that teams with a shared performance-approach goal orientation and high perceptions of their team abilities, will display motivated behavior and adaptive team regulation (rather than irrational delaying team actions), focused on task accomplishment and demonstration of team competence. When performance-approach goal oriented teams doubt their abilities, the achievement context is likely to be viewed as threatening, inducing anxiety and obtrusive thoughts, increasing the likelihood of engagement in team procrastination as a self-handicapping strategy. Therefore, we propose:

*Hypothesis 6:* The relationship of team performance-approach goal orientation and team procrastination is moderated by team efficacy, such that team performance-approach goal orientation is only positively related to team procrastination when team efficacy is low.

## Outcomes of Team Procrastination

Team effectiveness models characterize outcomes as “the results and by-products of team activity that are valued by one or more constituencies” (Mathieu et al., 2008, p. 412), including both performance and members’ affective reactions. Based on these models, we included both team performance and an indicator of team member’s affective reactions (i.e., stress) as important potential outcomes of team procrastination. Building on multilevel team motivation models (i.e., Chen and Kanfer, 2006; Chen and Gogus, 2008) we include both top–down effects of team processes on individual team-member’s states (i.e., team procrastination to individual stress) and bottom–up effects (e.g., the team’s average individual team members’ stress levels as predicting team performance).

Regarding team members’ *stress*, although procrastination may give some short-term relief and stress reduction (e.g., Tice et al., 2001), research has generally reported negative relationships between individual-level procrastination and mental health. For example, Tice and Baumeister (1997) showed that procrastinating students have better mental and physical health early in the semester, but worse health later on. Overall, procrastinators experienced more mental and physical health problems than non-procrastinators, suggesting an overall negative impact of procrastination on stress. Further, Sirois et al. (2003) found that procrastination among students relates to poorer health, treatment delay, higher levels of perceived stress, and fewer wellness behaviors. Similarly, Sirois (2014) found positive correlations of procrastination with perceived stress, and negative correlations with positive affect in two general adult samples.

Integrating individual-level theory and findings with multilevel conceptualizations of motivation in and of teams (e.g., Chen and Kanfer, 2006; Chen and Gogus, 2008), we expect top–down effects of team procrastination on individual team members’ well-being. Specifically, team members of procrastinating teams will feel heightened levels of anxiety and stress, especially toward the deadline or when the team has to perform the prepared tasks (i.e., before the oral debate).

*Hypothesis 7a:* Team procrastination is positively related to perceived stress among the individual team members.

Furthermore, we propose that team procrastination may interact with individual team-member characteristics in predicting stress among individual team members. We expect that team procrastination will especially result in heightened stress levels for individual team members that are generally not inclined to procrastinate (i.e., low trait procrastination). The rationale for this expectation is based on the person-environment fit literature, which suggests that compatibility between individuals and their environment (e.g., work group, organization) affects individual-level outcomes (e.g., Kristof-Brown et al., 2005). Specifically, team members low on trait procrastination likely experience supplementary misfit in teams high on team procrastination, having negative outcomes for the individual such as increased stress. Therefore, we expect:

*Hypothesis 7b:* Individual-level trait procrastination and team procrastination interact in the prediction of perceived stress among the individual team members, such that the relationship between team procrastination and individual-level stress is more positive for individuals with low rather than high levels of trait procrastination.

Lastly, regarding *performance*, previous theorizing suggested that procrastination may have both benefits and costs in terms of performance (e.g., Tice and Baumeister, 1997; Van Eerde, 2000). On the positive side, procrastination is believed to increase efficiency, to result in heightened motivation closer to the deadline, and to lead to more thinking time and creativity. On the negative side, procrastination may lead to increased time pressure, too little time to adequately complete the task, speed-accuracy trade-offs, and inability to deal with unforeseen obstacles or setbacks. Especially when procrastination leads to increased stress when the deadline approaches, task performance may suffer. In fact, we posit that such increased collective stress levels in teams may represent a powerful mechanism in the negative link between team procrastination and team performance.

In the present task context, teams had to perform an oral debate during which their performance as a team was rated. This represents a highly evaluative situation, because the teams had to conduct the debate for a grade in front of an audience and two assessors. Evaluative situations create test anxiety, which is detrimental for performance (Hembree, 1988). Even though performance was rated at the team level, the performance of individual team members during the debate is highly visible, which may further add to evaluation anxiety. We therefore propose that team procrastination will result in reduced team performance, in part because it evokes stress in the team. Specifically, because team procrastination leaves less time for optimally preparing the team tasks, coordinating the team’s inputs, and handling unexpected difficulties, stress levels in procrastinating teams are heightened. These heightened levels of collective stress in team may increase the likelihood that team members choke or collapse under the pressure, reducing their subsequent performance during the debate.

*Hypothesis 8:* Team procrastination is negatively related to team performance, and this relationship is mediated by collective stress in the team.

## Materials and Methods

### Task Context

To examine team procrastination and its predictors and outcomes, we selected a task setting in which multiple teams had to perform the same tasks within the same time frame with a set deadline. That is, to study procrastinatory behavior, a specified task-setting with a clear deadline is needed. Furthermore, because we were interested in team-member personality and team motivational state predictors rather than task-related predictors of team procrastination, and because we were interested in team performance, we opted for a setting in which the task was the same for all teams. Consistent with these criteria, the present study was conducted using a sample of undergraduate students enrolled in a debating course at a Dutch university.

The debating course consisted of three meetings which primarily took place in teacher-led classes of 10 to 12 students, to which students were randomly assigned. In the first course meeting, students received general instructions about debating and the course procedures in a plenary lecture. After that, within the classes debating teams were formed by the teacher and students based on the instruction to divide the class into four three-person teams (or one or two two-person teams when the class had 11 or 10 students). In the remainder of the first meeting the principles of debating were further taught and trained. For the second meeting, 1 week later, the teams collaborated on a written assignment (i.e., a debate between two parties on a given proposition which varied across teams). The collaborative work on the assignment included studying the topic, developing and selecting arguments, developing replies to the arguments, and writing all up in a paper containing a structured debate. The teams had to hand in their paper before a set deadline. During the second meeting, based on the papers, the teams practiced their debating skills and received feedback. After that, the teams were given a proposition and a position (pro or con) for which they had 1 week to jointly prepare arguments and practice for the oral debate. In the third meeting, 1 week later, there was a debating contest in which each team debated another team in front of an audience consisting of the other students. Two trained judges (who were blind to the study hypotheses, the teams’ performance during the course, and the teams’ scores on the study variables) independently graded each team’s debating performance.

### Participants and Procedure

To reduce common-method variance, survey data were collected at three points in time during the debating course (cf. Podsakoff et al., 2003). Time 0 measures involved individual characteristics (demographics and trait procrastination), and were collected 2 days before the first meeting (i.e., before teams were formed). Time 1 measures involved team efficacy, team goal orientation, and team procrastination concerning the written assignment. These measures were collected during the second meeting, when team members had been working together intensively for a week on their written assignment. Time 2 measures were collected shortly before the debating contest in the third meeting and involved team procrastination concerning the preparation of the oral debate and individual stress. Finally, team performance was reflected by the team’s grade for the oral debate (i.e., average of two judges).

Participation in the study was voluntary and students could discontinue their participation at any moment. Among participants gift cards of €50 and €15 were raffled. Data were treated strictly confidentially, which was emphasized in the survey instructions. Because the study involved no invasive or potentially harmful elements, it was declared exempt from further review by the department’s ethical committee.

Teams were included in the final sample only if at least two members provided valid data. As a result, the final sample consisted of 570 students divided over 209 debating teams (57 teams composed of two students and 152 teams composed of three students). Occasional missing responses occurred at the individual level (i.e., at Time 0, 1, and 2 respectively 4.9, 5.3, and 1.2% of the participants did not complete the questionnaire). Although there is debate on whether two-member constellations can be considered teams (see Williams, 2010), definitions of teams typically state that teams consist of two or more members (e.g., Salas et al., 1992; Ilgen, 1999). Even though some group phenomena may not apply to small groups, the focal concepts of the present study (i.e., team motivation, regulation, and performance) can be studied in small teams of two or three members (cf. Williams, 2010). Nevertheless, we conducted a team-level multivariate analysis of variance (MANOVA) with team size as factor and gender, age, and the study variables as dependents to test for differences between two- and three-member teams. The MANOVA showed some indication for differences between two-member and three-member teams, *F*(12,196) = 1.58, *p* = 0.10. Subsequent *t*-tests indicated that, on average, three-member teams scored a little higher on team performance-approach goal orientation, *M*_difference_ = 0.22, *t*(207) = 2.82, *p* < 0.01. We therefore control for team size in all our analyses.

Of the participants, 75.6% was female and the mean age was 21.01 (*SD* = 3.00). Most teams were women-only (*n* = 118) or mixed-gender teams (*n* = 76). Fifteen teams consisted only of men. In line with recommendations in the literature (Williams and Meân, 2004) we used a directional proportional measure to calculate team gender composition, that is, the proportion of men in the team. An overall team-level MANOVA with team gender composition as factor and age and the study variables as dependents indicated that teams differed significantly on the included variables depending on the team’s gender composition, *F*(22,394) = 2.23, *p* < 0.01. *Post hoc* Bonferroni tests showed that all-male teams scored higher than mixed-gender teams, and mixed-gender teams higher than all-female teams on team efficacy. We therefore control for team gender composition in all our analyses.

As data were collected over 3 years, a last overall team-level MANOVA was conducted to test whether mean differences existed between the 3 years on gender, age, and the study variables, indicating an overall significant effect of year, *F*(24,392) = 2.06, *p* < 0.01. Separate ANOVAs for each of the variables, only showed a significant effect of year for age, *F*(2,206) = 3.63, *p* < 0.05. We therefore controlled for average age of the team members in all our analyses.

Even though the assignment to classes was random, the composition of teams within the classes was not entirely random. We therefore included two questions at the end of the Time 2 questionnaire asking with how many of their team members students (a) had collaborated before, and (b) regularly interact with. The responses show that 367 respondents (64.4%) reported that they had collaborated with none of their team members before, and that 371 respondents (65.1%) reported that they regularly interacted with none of their team members. Since teams collaborated on team assignments and were graded as a team, they were highly interdependent, both in terms of task and outcome. To check this assumption, we assessed at Time 2 whether team members perceived interdependence at the debating task and its preparations (task interdependence; three items on a 5-point scale based on Campion et al., 1993) and whether they collaborated as a team on the debating task (cooperation; three items on a 5-point scale based on Van Mierlo and Kleingeld, 2010). Scores on task interdependence (*M* = 3.51, *SD* = 0.41) and cooperation (*M* = 3.96, *SD* = 0.43) suggest that teams indeed tended to agree with the task as being interdependent.

### Measures

The survey items at Time 0 (trait procrastination and demographics), Time 1 (team efficacy, team goal orientation, team procrastination), and Time 2 (team procrastination, individual stress) were administered at the individual level. Unless indicated otherwise, items were completed by using 5-point Likert scales ranging from 1 = *completely disagree* to 5 = *completely agree*. Team performance was assessed at the team level by the teams’ grade on the oral debate.

For team efficacy, team goal orientation, and team procrastination, we used a reference-shift model approach (Chan, 1998), which is the recommended approach to capture constructs that are conceptually defined in terms of shared perceptions of a team-level construct (cf. Klein et al., 2001; Van Mierlo et al., 2009). Specifically, individual team members responded to team-referent items for each measured construct, asking them to reflect on the team’s position on the construct of interest. Team-level scores were calculated by averaging the individual team-members’ scores per team. To assess the appropriateness of this aggregation procedure, we used the intraclass correlation *ICC1* (Bryk and Raudenbush, 1982) and the interrater agreement index *r*^∗^_wg(j)_ (Lindell et al., 1999). *ICC1* represents the proportion of variance in a dependent variable that is explained by team membership, and *r*^∗^_wg(j)_ indicates within-team agreement in terms of whether team members provided similar ratings on the construct in an absolute sense. In terms of interpretation, *ICC1*-values between 0.05 and 0.20 are considered typical (Bliese, 2000), and *r*^∗^_wg(j)_-values between 0.51 and 0.70 are considered to indicate moderate agreement and between 0.71 and 0.90 to indicate strong agreement (LeBreton and Senter, 2008).

#### Trait Procrastination

Individual participants’ general tendency to engage in procrastination was measured at Time 0 (before teams were composed) with nine items based on Lay’s (1986) General Procrastination Scale (GPS; e.g., “I generally delay before starting on work I have to do,” and “I often find myself performing tasks that I had intended to do days before”). In terms of the three factors underlying procrastination (Svartdal and Steel, 2017), and similar to the Pure Procrastination Scale (Steel, 2010), our scale represented implemental delay, decisional delay, and timeliness/lateness. However, as in the original GPS, the emphasis of the items is on implemental delay. Individual-level coefficient alpha was 0.84. For the analyses at the team level, the team’s average score of the individual team members’ levels of trait procrastination was considered as a configural team property (cf. Klein and Kozlowski, 2000).

#### Team Efficacy

Team efficacy was operationalized as task-specific collective efficacy beliefs, and was assessed at Time 1 with ten items based on Riggs and Knight’s (1994) personal efficacy beliefs scale. The items were adapted to refer to the specific task context of debating. In addition, consistent with the reference-shift model, the items were adapted such that they referred to individual team member perceptions of the team’s efficacy. Sample items include: “We believe that we are good debaters” and “There are some tasks required in debating that we as a team cannot do well” (reverse scored). Individual-level coefficient alpha for this scale was 0.83. Team-level scores were calculated by averaging the individual team-member’s scores per team. *ICC1* for team efficacy was 0.22, *F*(208,331) = 1.72, *p* < 0.001, and average *r*^∗^_wg(j)_ across teams was 0.75, thus supporting aggregation to the team level.

#### Team Goal Orientation

Team goal orientation was operationalized as the task-specific motivational orientation of the team, reflecting the purpose of achievement behavior in the specific setting of the debating course (cf. Dweck and Leggett, 1988; Elliot and McGregor, 2001; Harackiewicz et al., 2002). At Time 1, each of the four team goal orientations was measured with three items based on the Achievement Goal Questionnaire (Elliot and McGregor, 2001). The items were adapted such that they referred to the team (i.e., referent-shift model) and the specific debating context. Sample items include: “As a team, we want to learn as much as possible from preparing our debating assignments” for learning-approach, α = 0.73, *ICC1* = 0.10, *F*(208,331) = 1.30, *p* < 0.05, average *r*^∗^_wg(j)_ = 0.78, “It is important for my team to do better in the debating presentation than the other teams” for performance-approach, α = 0.82, *ICC1* = 0.24, *F*(208,331) = 1.84, *p* < 0.001, average *r*^∗^_wg(j)_ = 0.73, “Sometimes, we are concerned that we do not understand the content of our debating assignments as fully as we would like” for learning-avoidance, α = 0.74, *ICC1* = 0.24, *F*(208,331) = 1.81, *p* < 0.001, average *r*^∗^_wg(j)_ = 0.72, and “Our goal in the debating presentation is to avoid performing poorly compared to other teams” for performance-avoidance, α = 0.73, *ICC1* = 0.05, *F*(208,331) = 1.15, *p* = 0.14, average *r*^∗^_wg(j)_ = 0.67. In summary, *ICC1* and *r*^∗^_wg(j)_-values for the team goal orientation measures support aggregation to the team level for learning-approach, performance-approach, and learning-avoidance. The results for team performance-avoidance were less supportive and cast some doubt about the shared nature of team performance-avoidance goal orientation in our sample. Team-level scores for the goal orientations were calculated by averaging the individual team-member’s scores per team.

#### Team Procrastination

Procrastinatory behavior of the team was assessed both at Time 1 and Time 2 with four items. The items were based on individual-level behavioral procrastination measures used in previous research (e.g., Solomon and Rothblum, 1984; Milgram and Toubiana, 1999; Wolters, 2003) and Steel’s (2007) behavioral definition of procrastination to ensure content validity. In order to be able to measure procrastinatory behavior, the items were framed as referring to the specific task at hand (rather than to a general tendency). Furthermore, consistent with the reference-shift approach, items were framed as referring to the individual team member’s perceptions of procrastinatory behavior of the team. Thus, the items at Time 1 referred to the team ‘working on the written assignment,’ whereas the items at Time 2 referred to the team ‘preparing for the oral debate.’ The specific items for Time 2 team procrastination were: “Despite our intentions to start timely with the preparations for the oral debate, we had to do a lot still at the last moment,” “We often postponed working on our preparation of the oral debate,” “When we planned to work on preparing for the oral debate, we often engaged in other things (e.g., surfing the internet, chatting about other things, etc.),” and “Our team postponed the preparations for the oral debate until the last minute.” Individual-level coefficient alpha was 0.72 at Time 1 and 0.73 at Time 2. Because one of the main goals of the present study was to examine whether team members have a shared conception of the team’s collective procrastinatory behavior, we report the aggregation analyses in the results section.

As a check of the validity of our team procrastination measure, we retrieved the time at which the teams submitted their written assignment via the electronic learning environment. We calculated the team’s timeliness as the number of seconds left between the submission time and the deadline time (i.e., higher scores indicate more timely completion of the team assignment). Although low timeliness is not equivalent to procrastination because last minute completion could have multiple causes, procrastinating teams by definition should be less timely in finishing tasks. Supporting the validity of our team procrastination measure, Time 1 team procrastination correlated negatively with timeliness, *r* = -0.36, *p* < 0.01.

#### Stress

Individual team-members’ stress was measured with the abbreviated 7-item stress subscale of the Depression Anxiety Stress Scale (Lovibond and Lovibond, 1995), adapted to the present study context. A sample item is: “I found it difficult to relax during this course,” and “I found myself getting agitated during this course.” Individual-level coefficient alpha for this scale was 0.86. The analyses with stress as the outcome variable were performed with multilevel regression analysis, using individual-level scores of team-member stress. The analyses with team performance as the outcome variable were performed at the team level (because team performance has no variance at the individual level), using average team-member stress scores per team. Supporting the use of averaged team-member stress scores, the *ICC1* was 0.15, *F*(208,351) = 1.48, *p* < 0.001, and the *r*^∗^_wg(j)_ was 0.61.

#### Team Performance

Prior to the third course meeting, the teams were assigned to a larger class consisting of six to eight teams. The teams performed a debate on an assigned proposition with a position pro or con. In each class, two trained judges (who were blind to the study purposes) independently assessed each team’s performance. The grading procedure was standardized and based on a training and detailed assessment instructions. On a scale from 1 to 10, judges rated each team’s performance on five core elements of debating: argumentation, countering, presentation, teamwork, and interruptions. These elements represent standard performance criteria in debating contests and all teams were familiar with the rating procedure. The ratings on the five elements were combined into one weighted performance score per judge, using the following weights: 40% argumentation, 20% countering, 20% presentation, 10% teamwork, and 10% interruptions. The weighted performance scores of the two judges were averaged to derive our measure of team performance. The judges’ weighted performance scores were strongly related, indicating sufficient interrater reliability, *r* = 0.68, *p* < 0.001.

## Results

### Procrastination as a Team-Level Construct

To examine whether team procrastination exists as a team-level construct, we first calculated the *ICC1*-values and *r*^∗^_wg(j)_-values for Time 1 and Time 2 team procrastination. Team membership accounted for considerable and significant variance in both Time 1 team procrastination, *ICC1* = 0.62, *F*(208,331) = 5.13, *p* < 0.001, and Time 2 team procrastination, *ICC1* = 0.36, *F*(208,352) = 2.52, *p* < 0.001. Average *r*^∗^_wg(j)_ across teams was 0.75 for Time 1 and 0.71 for Time 2. Taken together, these findings suggest that team members share a common perception of the procrastinatory behaviors in their team. Aggregated team-level scores on team procrastination at Time 1 varied between 1.00 and 4.25 (*M* = 2.48, *SD* = 0.63) and scores on team procrastination at Time 2 varied between 1.00 and 3.88 (*M* = 2.38, *SD* = 0.52), indicating that teams differ in their procrastinatory behavior.

As displayed in **Table 1**, the correlation between Time 1 and Time 2 team procrastination was 0.42 (*p* < 0.001), suggesting that teams who procrastinated on their written assignment were also more likely than other teams to procrastinate on the preparation of the oral debate. This finding suggests that team procrastination may be interpreted as a team-level behavioral construct that has some stability over time.

**Table 1:** Team-level means, standard deviations, and correlations between the study variables.

	*M*	*SD*	1	2	3	4	5	6	7	8	9	10	11	12
**Time 0 variables:**											
(1) Team size^a^	2.73	0.45												
(2) Team gender composition^b^	0.24	0.32	0.06											
(3) Average age of team members	21.08	2.09	-0.11	0.15*										
(4) Team member trait procrastination	3.24	0.42	-0.08	0.07	-0.04									
**Time 1 variables:**											
(5) Team efficacy	2.97	0.34	0.09	0.31**	0.12	-0.08								
(6) Team learning-approach GO	3.56	0.37	0.00	-0.10	0.14*	-0.09	0.18**							
(7) Team performance-approach GO	3.07	0.51	0.19**	0.12	0.10	-0.05	0.43**	0.49**						
(8) Team learning-avoidance GO	2.72	0.49	-0.08	-0.19**	0.06	0.13	-0.36**	0.15*	0.03					
(9) Team performance-avoidance GO	3.06	0.43	0.13	-0.14*	0.07	-0.06	-0.03	0.35**	0.47**	0.37**				
(10) Team procrastination	2.48	0.63	-0.04	0.06	0.02	0.22**	-0.09	-0.06	0.04	0.29**	0.10			
**Time 2 variables:**											
(11) Team procrastination	2.38	0.52	-0.03	0.10	-0.06	0.21*	-0.13	-0.21**	-0.06	0.22**	-0.05	0.42**		
(12) Team member stress	2.55	0.49	-0.10	-0.17*	-0.07	0.14*	-0.39**	0.01	-0.07	0.32**	0.08	0.11	0.17*	
**Performance variables:**											
(13) Team performance (debate)	7.35	0.51	0.09	-0.01	-0.01	-0.09	0.15*	-0.01	0.12	-0.20**	0.01	0.01	0.02	-0.17*

*The means, standard deviations, and correlations are based on team-level (i.e.,aggregated) data. N = 209 teams. GO, goal orientation. ^a^Team size varied between 2 and 3 team members. ^b^Team gender composition ranged from 0 = all-female to 1 = all-male. Variables 4–12 were answered on Likert scales ranging from 1 to 5. Variable 13 was graded on a scale from 1 to 10. ^∗∗^p < 0.01. ^∗^p < 0.05.*

### Predictors of Team Procrastination

Hypotheses 1–6 concern the prediction of team procrastination as outcome variable. Because this outcome is specified at the team level and thus only has variance at the team level, we used regular regression analysis at the team level. **Table 2** presents the regression results predicting team procrastination, controlling for team size (two-member vs. three-member teams), gender composition of the team, and average age of the team members. Average levels of team members’ trait procrastination (Time 0) positively predicted team procrastination on both the written assignment (Time 1), β = 0.22, *t*(204) = 3.18, *p* < 0.01, and the preparation for the oral debate (Time 2), β = 0.20, *t*(204) = 2.88, *p* < 0.01. Thus, in support of Hypothesis 1, teams with members who score, on average, higher on individual trait procrastination, consistently engaged in more team procrastinatory behavior over the course of time. We further explored whether other compositional operationalizations of team member trait procrastination contributed to the explanation of team procrastination, but neither within-team standard deviation, nor the minimum or maximum team-member score added significantly to the prediction of team procrastination beyond the team mean trait procrastination score.

**Table 2:** Regression analysis of team procrastination on demographics, trait procrastination, team efficacy, and team goal orientation.

Predictor	Time 1 team procrastination (β)	Time 2 team procrastination (β)		
		Step 1	Step 2	Step 3
**Time 0 variables:**				
Team size^a^	-0.03	-0.03	-0.01	-0.01
Team gender composition^b^	0.04	0.10	0.12^†^	0.12^†^
Average age of team members	0.02	-0.07	-0.05	-0.06
Composition trait procrastination	0.22**	0.20**	0.14*	0.15*
**Time 1 variables:**				
Team efficacy			-0.07	-0.06
Team learning- approach GO			-0.23**	-0.24**
Team performance- approach GO			0.12	0.09
Team learning- avoidance GO			0.26**	0.27**
Team performance- avoidance GO			-0.09	-0.07
**Interaction:**				
Team efficacy × Team performance- approach GO				0.13*
Δ *R*^2^			0.10**	0.02*
Multiple *R*	0.23*	0.24*	0.40**	0.42**
Adjusted *R*^2^	0.03	0.04	0.12	0.13

*N = 209 teams. GO, goal orientation. ^a^Team size varied between 2 and 3 team members. ^b^Team gender composition ranged from 0 = all-female to 1 = all-male. ^∗∗^p < 0.01. ^∗^p < 0.05. ^†^p < 0.10.*

The team motivational states (i.e., team efficacy and team goal orientation) were measured at Time 1 after the teams had been working together for a week. To test whether these motivational states predict team procrastination, they were added to the regression of Time 2 team procrastination. As displayed in **Table 2**, team motivational states explained significant additional variance in team procrastination beyond team size, gender composition, average team member age, and compositional trait procrastination, Δ*R*^2^ = 0.10, *F*(5,199) = 4.73, *p* < 0.001, further indicating that team procrastination is a distinct team-level phenomenon depending on team-level state predictors. In contrast to Hypothesis 2, team efficacy did not predict team procrastination, β = -0.07, *t*(199) = -0.84, *p* = 0.40. Regarding team goal orientation, the results support Hypothesis 3, indicating that teams with a learning-approach goal orientation were less likely to procrastinate on preparing for the oral debate, β = -0.23, *t*(199) = -2.92, *p* < 0.01. Hypothesis 4 and 5 stated that both avoidance goal orientations would positively relate to team procrastination. In support of Hypothesis 4, results demonstrate that team learning-avoidance goal orientation positively related to team procrastination, β = 0.26, *t*(199) = 3.42, *p* < 0.001. However, team performance-avoidance goal orientation did not predict team procrastination, β = -0.09, *t*(199) = -1.13, *p* = 0.26 (Hypothesis 5 not supported).

**FIGURE 2:**
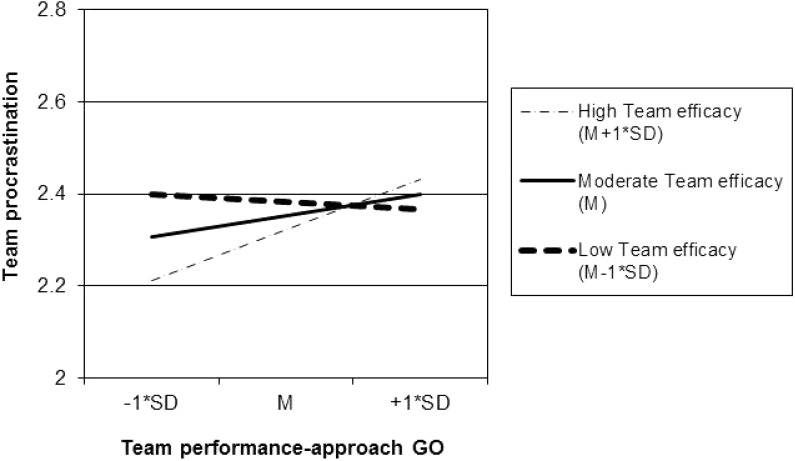
Simple regression slopes of team procrastination on team performance-approach goal orientation (GO) for low (*M* – 1^∗^*SD*), moderate (*M*), and high (*M* + 1^∗^*SD*) levels of team efficacy.

Hypothesis 6 stated that team performance-approach goal orientation and team efficacy would interact in predicting team procrastination. In Step 3 of the regression analysis as displayed in **Table 2**, the interaction between performance-approach goal orientation and team efficacy was added (using centered scores for all independent variables). The interaction explained significant additional variance in team procrastination, Δ*R*^2^ = 0.02, *F*(1,198) = 3.99, *p* < 0.05. The form of the interaction was further analyzed following procedures recommended by Aiken and West (1991). **Figure 2** displays the slopes of team performance-approach goal orientation – team procrastination relationship at three levels of team efficacy (i.e., 1 SD above the mean, the mean, and 1 SD below the mean). Contrary to Hypothesis 6, the performance-approach goal orientation – team procrastination relation was significantly positive at high levels of team efficacy, *B* = 0.21, *t*(206) = 2.03, *p* < 0.05, but not significantly different from zero at low levels of team efficacy, *B* = -0.03, *t*(206) = -0.29, *p* > 0.05. In other words, performance-approach goal orientation positively predicted team procrastination, but only for teams high (rather than low) on team efficacy.

### Outcomes of Team Procrastination

**FIGURE 3:**
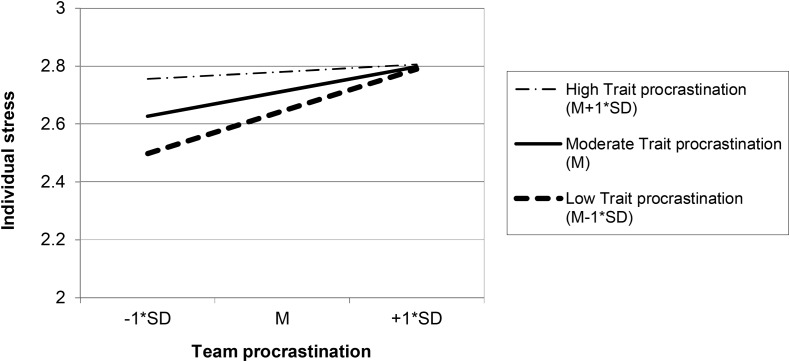
Simple regression slopes of individual-level stress on team procrastination for low (*M* – 1^∗^*SD*), moderate (*M*), and high (*M* + 1^∗^*SD*) levels of individual-level trait procrastination.

Hypotheses 7a and 7b concern the relationship of team procrastination with team-member stress. Because this outcome is measured at the individual level with individuals (Level 1) nested in teams (Level 2), we used multilevel regression analysis (i.e., with the mixed model procedure in SPSS). Furthermore, because team-member stress was assessed at Time 2 shortly before performing the oral debate, we tested Hypotheses 7a and b for team procrastination at Time 2, which also reflected the oral debate. We controlled for Time 0 trait procrastination to rule out the possibility that the team procrastination – stress relation is spurious, caused by pre-existing levels of trait procrastination among the team members. **Table 3** presents the results. In Model 1, Time 2 individual-level stress was regressed on individual-level trait procrastination (Time 0) and team procrastination (Time 2), controlling for team size, individual gender, and individual age. Females and individuals high on trait procrastination were more likely to perceive stress than males and those low on trait procrastination. Further, in support of Hypothesis 7a, the findings demonstrate a significant positive relationship of team procrastination with stress among individual team members. In Model 2, the cross-level interaction between Time 0 individual-level trait procrastination and Time 2 team procrastination was added. The results demonstrate a negative parameter estimate, *B* = -0.172, *SE* = 0.088, which was significant at 0.10 and approached significance at.05, *t* = -1.945, *p* = 0.052. Regression lines for high (i.e., *M* + 1 *SD*), mean, and low (i.e., *M* - 1 *SD*) levels of trait procrastination are plotted in **Figure 3**. These findings indicate that, in line with Hypothesis 7b, team procrastination related positively to individual-level stress especially for team members low on trait procrastination. Team procrastination related less strongly to individual-level stress for team members high on trait procrastination.

**Table 3:** Multilevel regression analysis of individual-level stress on team procrastination, individual-level trait procrastination, and their cross-level interaction.

Predictor	Time 2 individual-level stress			
	Model 1		Model 2	
	Estimate	*SE*	Estimate	*SE*
**Fixed part:**				
Intercept	1.914**	0.382	0.675	0.744
Control variables:				
Team size^a^	-0.080	0.081	-0.085	0.080
Individual gender^b^	-0.212**	0.071	-0.206**	0.070
Individual age	0.005	0.009	0.006	0.009
Predictors:				
Individual trait procrastination	0.104*	0.044	0.510*	0.213
Time 2 team procrastination	0.188**	0.067	0.719*	0.281
Cross-level interaction (Individual trait procrastination × Time 2 team procrastination)			-0.172^†^	0.088
**Random part:**				
Residual	0.384**	0.029	0.384**	0.029
Individual trait procrastination	0.008**	0.002	0.007**	0.002
2 Log Likelihood	1105.692		1101.936	
Akaike’s Information Criterion (AIC)	1121.692		1119.936	

*^a^Team size varied between 2 and 3 team members. ^b^0 = female, 1 = male. Due to occasional missing values N_individuals_ = 539 and N_teams_ = 208. ^∗∗^p < 0.01. ^∗^p < 0.05. ^†^p < 0.10.*

Hypothesis 8 stated that team procrastination relates negatively to team performance, as mediated by collective stress in the team. Because the outcome of team performance is rated at the team level and thus has no variance at the individual level, we used regular regression analysis at the team level. Team performance was rated by different pairs of judges (i.e., each class consisting of 6–8 teams had a different combination of two judges). Across the dataset, there were 22 different judge pairs, with each judge pair rating the performance of 6–17 teams (*M* = 9.45; *SD* = 2.64). In other words, the team performance ratings were nested within judge pair. Because performance ratings are usually strongly affected by rater effects (e.g., Scullen et al., 2000), we controlled for judge pair in the analyses of team performance. Specifically, we computed 21 dummy variables to indicate each of the 22 judge pairs, and included these dummies in the regression analysis to partial out the variance that can be attributed to the judge pairs (and thus likely reflects rater effects rather than the team’s performance).

**Table 4** demonstrates the results of the regression analysis of team performance on Time 2 team procrastination and Time 2 average team member stress, controlling for team size, gender composition, average age of the team members, average trait procrastination, and judge pair (using the 21 dummies). Team procrastination did not explain significant unique variance in team performance beyond the control variables, Δ*R*^2^ = 0.00, *F*(1,181) = 0.13, *p* > 0.05, but average team member stress did, Δ*R*^2^ = 0.04, *F*(1,180) = 8.73, *p* < 0.01. The negative beta-weight (β = -0.21) indicates that the more stress the team members had, the lower their team’s performance ratings on the oral debate. To test the indirect effect of team procrastination on team performance through average team member stress, we used Hayes’ (2013) PROCESS macro to conduct a regression-based bootstrap analysis. In support of Hypothesis 8, the results (using 10,000 bootstrap samples with replacement) indicated a significant indirect effect of team procrastination via average team member stress on team performance of -0.034, SE = 0.021, 95% CI [-0.084,-0.001].

**Table 4:** Regression analysis of team performance on average team member stress and team procrastination.

Predictor	Team performance on the oral debate (β)		
	Step 1	Step 2	Step 3
**Control variables:**
Team size^a^	0.07	0.07	0.06
Team gender composition^b^	0.03	0.03	-0.01
Average age of team members	0.01	0.01	-0.01
Composition trait procrastination	-0.11	-0.11	-0.09
Judge pair dummies	(21 beta-weights omitted from display in the table for reasons of clarity)
**Step 2:**
Time 2 team procrastination		-0.03	0.01
**Step 3:**
Time 2 average team member stress			-0.21**
Δ *R*^2^		0.00	0.04**
Multiple *R*	0.48**	0.48**	0.52**
Adjusted *R*^2^	0.12	0.12	0.16

*N = 208 teams. ^a^Team size varied between 2 and 3 team members. ^b^Team gender composition ranged from 0 = all-female to 1 = all-male. ^∗∗^p < 0.01.*

## Discussion

The present study introduced the concept of procrastination to the team motivation literature. Team procrastination is characterized as an instance of failing self-regulation of the team, defined as the unplanned, voluntary, and irrational delay of intended goal-directed team activities. The results supported the existence of team procrastination as a team-level construct that has some stability over time. Team procrastination was predicted by characteristics of the individual team members (i.e., trait procrastination), but also by teams’ shared motivational states (i.e., team learning-approach goal orientation, team learning-avoidance goal orientation, and the interaction between team efficacy and team performance-approach goal orientation). In terms of outcomes, the findings indicate that team procrastination relates to increased levels of stress, somewhat more so for team members low on trait procrastination. Furthermore, team procrastination indirectly related to lower team performance through the increased levels of stress it evoked in the team.

### The Concept of Team Procrastination

The present study findings indicate that procrastination exists at the team level, such that teams as a collective may engage in procrastination of team tasks. Team members were found to hold a shared perception of the procrastinatory behaviors of their team, and this perception was showed some stability across the two time points and tasks. Thus, in terms of Klein and Kozlowski’s (2000) classification of team constructs, we defined team procrastination as a shared team property, and found empirical support for this sharedness. Our findings extend descriptive models of team development (e.g., Gersick, 1988, 1989; Chang et al., 2003) by suggesting that teams may differ in their pacing depending on their level of team procrastination. Our findings also add to previous research on team regulation (e.g., Marks et al., 2001; DeShon et al., 2004; Chen et al., 2005; Kozlowski and Ilgen, 2006; Rapp et al., 2014) by introducing a new construct indicative of failing team regulation. Whereas previous studies have demonstrated the existence and importance of team-level regulatory constructs such as team planning (e.g., Gevers et al., 2009; Mehta et al., 2009) and team monitoring (e.g., Marks and Panzer, 2004; Chen et al., 2005), the present findings suggest that team regulation can also fail as indicated by team procrastination. As such, team procrastination seems a promising team process construct, indicating a failure to turn team goals and plans into actual actions.

In the current study we defined and operationalized team procrastination as collective behavior (or lack thereof) of the team. Similar to the distinction between trait procrastination and procrastination as a behavior in the individual-level literature (e.g., Steel, 2007), future research is needed to investigate if and under what circumstances team procrastination can develop into a more stable team climate or shared mindset of a team, much like a team-level “trait” (cf. Hofmann and Jones, 2005). The substantial stability over time of the team procrastination scores in the current study do seem to point in this direction. These were, however, temporary teams on a short-term project. It would be interesting to see to what extent the observed patterns generalize to ongoing teams over longer timespans. Also, based on team development models (e.g., Gersick, 1988, 1989; Chang et al., 2003) research may seek to examine during which team stages team procrastination is more or less likely, and whether team procrastination predicts temporal awareness in teams (cf. Steel and König, 2006). Furthermore, future research is needed to examine how procrastinatory behavior of individual team members affects or interacts with other team members’ cognitions and behavior, and has bottom–up effects on team processes (e.g., team motivational states, team behavior).

### Antecedents of Team Procrastination

In terms of antecedents, the present findings indicate that team procrastination is predicted both by characteristics of individual team members and by teams’ shared motivational states. Extending previous research on individual team member traits (i.e., configural team properties) as predictors of team processes and emergent states (e.g., Barrick et al., 1998; Van Mierlo and Van Hooft, 2015), we found support for the idea that teams that are composed of team members who score low on trait self-regulation (i.e., high trait procrastination), more likely engage in team procrastination. This relationship can be explained such that team members high on trait procrastination more likely set norms, socialize in their teams, and socially interact with their team members in a way that promotes the team’s irrational delay of intended goal-directed team activities. However, future research should further test such explanatory mediating mechanisms.

Other compositional measures such as the standard deviation, lowest score, or highest score on trait procrastination among the team members did not add to the prediction of team procrastination. Because, in the current study, this was an exploratory excursion rather than our main focus, more research is needed to investigate the effect of team composition on team procrastination. Based on the relative contributions model (cf. Mathieu et al., 2014), future research could examine if and to what extent specific individual team members may have a disproportional influence on team procrastination, for example, due to individual team member’s status or prototypicality for the team.

Further, it should be noted that the effect size of the relationship between trait procrastination and team procrastination was relatively modest. This finding parallels the individual-level procrastination literature, which reports only moderate correlations between trait procrastination and procrastinatory behavior (e.g., Solomon and Rothblum, 1984; Lay, 1986; Ferrari, 1993; Tice and Baumeister, 1997; Ferrari and Tice, 2000; Van Eerde, 2003). Our and previous findings thus seem to indicate that actual procrastinatory behavior (both at the individual and team level) is determined not only by dispositional factors but also, and to a considerable extent, by situational factors and temporary states.

In the present study, we examined five team motivational states as potential predictors of team procrastination. First, contrary to our expectations, team efficacy did not relate to team procrastination. This finding is inconsistent with the individual-level literature that has found relatively strong negative correlations between self-efficacy and procrastination (see meta-analysis by Steel, 2007), although it is important to note that these findings are mostly based on trait rather than behavioral measures of procrastination. A potential explanation may be that the relationship between team efficacy and team procrastination is not always negative, depending on context, task perceptions, and level of analysis. For example, research on individual-level self-efficacy has suggested that its role in predicting resource allocation and performance is more complex, with between-individual studies demonstrating positive relationships (Stajkovic and Luthans, 1998) and within-individual studies demonstrating positive, negative, or non-monotonic relationships (e.g., Vancouver and Kendall, 2006; Vancouver et al., 2008; Seo and Ilies, 2009). Also, research on the role of team efficacy in predicting team performance has demonstrated that the generally positive relationship between team efficacy and performance (e.g., Gully et al., 2002; Stajkovic et al., 2009) may under some conditions be curvilinear, where high levels of team efficacy relate to low performance, especially for teams with low levels of team monitoring (Rapp et al., 2014). Although procrastination is typically different from planned effort and resource allocation (as it refers to *unplanned* voluntary delay), future research should examine the role of team efficacy in dynamic study settings allowing for a distinction between between-teams effects and within-teams effects over time.

Second, team learning-approach goal orientation was found to negatively predict team procrastination. Teams high on learning-approach goal orientation are thus less likely to engage in maladaptive escapist behaviors and avoidant coping as indicated by collective procrastination. This finding is in line with individual-level studies on trait procrastination (Scher and Osterman, 2002; Wolters, 2003, 2004; Howell and Watson, 2007; Howell and Buro, 2009; Seo, 2009; Corkin et al., 2011). Also, team-level research on team motivation and regulation has generally supported the adaptive role of a team learning-approach goal orientation (e.g., DeShon et al., 2004; Mehta et al., 2009; Van Mierlo and Van Hooft, 2015). Practically, these findings suggest that teams may benefit from instilling a learning-goal orientation. A learning-approach goal orientation can be promoted by having leaders focus on providing feedback, rewarding effort, encouraging experimentation and learning from failures, and assigning difficult tasks (Dragoni and Kuenzi, 2012), while establishing a safe and trustful interpersonal environment. Also, the individual-level literature has demonstrated that situational cues and brief trainings sessions (e.g., setting mastery goals, instructing group members to provide developmental feedback, viewing errors as learning opportunities, encouraging using different strategies) can endorse a learning goal orientation in individuals (e.g., Linnenbrink, 2005; Noordzij et al., 2013). However, further research is needed to examine the applicability of such interventions to the team-level.

The third and fourth team motivational state that we included both concern avoidance goal orientations, that is, team learning-avoidance and team performance-avoidance. Based on previous theory and research (e.g., Elliot, 1999), both avoidance goal orientations were expected to positively predict team procrastination. Avoidance goal orientations focus on failure, leading to avoidant regulation strategies in order to prevent negative outcomes. However, this prediction was only supported for team learning-avoidance goal orientation. The positive relationship of team learning-avoidance goal orientation with team procrastination aligns with previous findings in the individual-level procrastination literature (Scher and Osterman, 2002; Howell and Watson, 2007; Howell and Buro, 2009; Seo, 2009; Corkin et al., 2011). Future research is needed to examine the underlying explanatory mechanisms in more detail. For example, teams with a learning-avoidance goal orientation may have heightened levels of worry, increased preoccupation with risk, a strong focus on preventing mistakes, and overly detailed deliberations before any action is taken (cf. Elliot and McGregor, 2001; Van Mierlo and Van Hooft, 2015), which may result in decisional and behavioral delays and postponement.

The predicted positive relationship of team performance-avoidance goal orientation and team procrastination was not supported. One explanation for this lack of findings may relate to the less convincing results regarding the sharedness of this team state. However, individual-level studies have also not always found support for the predicted positive relationship between performance-avoidance goal orientation and procrastination (e.g., Scher and Osterman, 2002; Howell and Watson, 2007; Howell and Buro, 2009; Corkin et al., 2011). In addition, various studies on team motivation and regulation reported a lack of support regarding the negative role of team performance-avoidance goal orientation for adaptive team processes (e.g., Mehta et al., 2009; Van Mierlo and Van Hooft, 2015). The task context may provide another possible explanation. That is, our study concerned the goal-striving system (rather than the goal-choice system). Because teams had to perform the given tasks as a team, withdrawal was not a real option (less likely even for individuals in a team as they would not only harm themselves but also their team), and the deadline was hard (i.e., performing an oral debate on a set date and time). In such conditions, a focus on preventing demonstrating incompetence might possibly not be as harmful for team motivation and regulatory processes.

Lastly, the role of team performance-approach goal orientation in predicting team procrastination was found to depend on team efficacy. However, the form of the interaction ran contrary to our expectations, such that team performance-approach goal orientation related positively to team procrastination, but only for teams high rather than low on team efficacy. In other words, especially teams with high efficacy that were focused on demonstrating their competence were vulnerable to procrastination of team tasks. This finding may be explained from a control theory perspective (e.g., Carver and Scheier, 1982; Vancouver and Kendall, 2006). That is, highly efficacious teams with a strong performance-approach goal orientation more likely evaluate their goals as easy, requiring less effort and regulatory strategies than anticipated. For such teams, high performance with low effort is an ideal scenario, because it allows them to demonstrate competence or even superiority. Such a setting will leave ample room for team procrastination, with teams investing less in deliberate self-regulation strategies and being more open to distractions, even if they had originally planned to work on the task. For teams low on performance-approach goal orientation, in contrast, the role of team efficacy aligns with social cognitive theory (e.g., Bandura, 1982; Stajkovic and Luthans, 1998) and individual-level procrastination research (e.g., Steel, 2007) in that team efficacy in these teams negatively predicted team procrastination: the more efficacy, the less procrastination.

### Consequences of Team Procrastination

We focused on both individual-level (i.e., perceived stress among team members) and team-level (i.e., team performance) outcomes of team procrastination. The results demonstrate that team-level behavior in terms of procrastination positively related to individual team members’ stress levels. This finding extends previous research on trait procrastination and well-being (e.g., Tice and Baumeister, 1997; Sirois et al., 2003; Sirois, 2014), indicating that stress is not only predicted by people’s own procrastination but also by their teams’ procrastination. In line with person-environment fit theory (e.g., Kristof-Brown et al., 2005) our multilevel analyses showed some tentative support for the idea that the relationship of team procrastination with stress is more pronounced for team members with low levels of trait procrastination. Although the cross-level interaction only approached significance, this finding suggests that individual and team-level characteristics interact to determine individual-level outcomes.

Further, our findings demonstrate mixed support for the role of team procrastination in predicting team performance. That is, team procrastination was not significantly (cor)related to team performance, but did have an indirect negative relationship with team performance, through the team’s collective stress levels. This finding of an indirect *negative* relationship between team procrastination and performance in absence of a significant overall relationship suggests that there may be other indirect *positive* relationships through untested intermediate variables that neutralize the observed indirect negative link. For example, team procrastination may have led to more bonding in the team or more room for creative ideas, which may in turn have boosted team performance. The notion that procrastination may have both costs and benefits is also regularly coined in the individual-level procrastination literature (e.g., Tice and Baumeister, 1997; Van Eerde, 2000, 2003), although cumulative evidence is more supportive of its negative effects (Steel, 2007). Nevertheless, future research is needed to further examine both positive and negative consequences of team procrastination, in terms of interpersonal team processes (e.g., cohesion, bonding, conflict) as well as team tasks (e.g., creativity, decision-making, quality of preparation).

## Limitations and Conclusion

As with all research, our findings and conclusions should be interpreted in the context of several boundary conditions and limitations. First, our study focused on small groups of three (or sometimes two) members. However, our group size was defined by the context of debating (i.e., debating teams typically consist of two to three members) and can therefore be considered as ecologically valid. Also, definitions typically describe groups and teams as collectives composed of two or more individuals, who interact, are interdependent, and have a shared goal (e.g., Salas et al., 1992; Ilgen, 1999), which was the case in the present study. Nevertheless, group dynamics in small groups differ from those in larger groups, and our conclusions are limited to small groups. Therefore, future research is needed to examine the validity of our findings for larger teams.

Second, we focused on teams in an educational setting, which may limit the possibility to generalize our findings to, for example, business settings. Procrastination may be less prevalent in employee samples than in student samples, although it has also been shown to occur regularly among adults (e.g., Ferrari et al., 1995, 2007). We opted for this sample because it provided some important controls, while at the same time having real teams engaging in real tasks with real and substantial consequences. For example, we were able to measure individual trait procrastination before the teams were formed. Therefore, it is unlikely that the relationship of trait procrastination with team procrastination and the sharedness of team procrastination are caused by attraction and selection processes during team formation (cf. Dragoni and Kuenzi, 2012). Also, because the team task and performance requirements were identical for all teams (i.e., a written assignment and an oral debate with set deadlines), we were able to accurately measure team procrastination and team performance.

Third, our measure of team procrastination was newly developed, although carefully based on previous individual-level measures of procrastinatory behavior and the definition of team procrastination. Future research is needed to further investigate and validate the construct of team procrastination. In addition, most of our measures relied on self-report, which may yield common method bias. However, we sought to reduce this threat by temporal and proximal separation of predictor and criterion measures (cf. Podsakoff et al., 2003). In addition, team procrastination related significantly to a non-self-report measure of timeliness, and our criterion measure of team performance was obtained from trained observers.

A fourth limitation may be the use of a correlational design, which restricts the possibility to draw empirically based causal conclusions. Although reverse causality is unlikely because of the temporal separation of our constructs and use of different sources, causality can still only be assumed on theoretical grounds because of the possible role of third variables. Further experimental research is needed to empirically test for causal effects (e.g., motivational states on team procrastination, team procrastination on stress). In addition, future research can investigate whether it is possible to manipulate team procrastination, building on previous experimental research on individual-level procrastination (e.g., Ferrari and Tice, 2000).

Lastly, our conceptual model and study focused on the role of team-member characteristics and team motivational states in predicting team procrastination and subsequent team outputs (i.e., member’s affective reactions and team performance). Models of team effectiveness (e.g., IMOI; Ilgen et al., 2005; Mathieu et al., 2008) suggest that team outputs may influence team motivational states and processes in the next performance episode. Because the present study followed project teams during their entire life cycle, with measurements before the teams were formed up to team performance ratings at the end, and teams were dissolved after the completion of the project, we were not able to examine such feedback effects from one episode to the next. Future research may take a more dynamic approach to investigating team procrastination during multiple performance episodes, allowing for examination of the role of team outputs in predicting subsequent team procrastination.

In conclusion, the present study introduced the concept of procrastination to the team motivation literature, showing that some teams procrastinate, and that collective procrastination relates to increased levels of stress among the team members, which may lower the team’s performance. Team procrastination was found to depend not only on characteristics of the individual team members, but also on the teams’ shared motivational states. Regardless of the study limitations, we believe that these findings add to the theoretical understanding of self-regulatory processes of teams, and highlight the practical importance of paying attention to team-level states and processes such as team goal orientation and team procrastination.

## Ethics Statement

The authors conformed to ethical guidelines for data collection, processing, and storage as prescribed by APA, the Netherlands Institute for Psychology, and the Erasmus University Rotterdam. Informed consent was obtained from all participants. Participation was voluntary and participants could discontinue their participation in the study at any moment. Data were treated strictly confidentially. The study involved no elements that could be qualified as invasive or potentially harmful. Based on these features, the study was exempt from review by the ethical committee of the Department of Psychology, Education, and Child Studies at the Erasmus University Rotterdam.

## Author Contributions

EVH: developing the study idea, designing the study, developing materials, coordinating data collection, data management, data analysis, and writing the paper. HVM: developing the study idea, designing the study, developing materials, coordinating data collection, data management, parts of data analysis, providing comments, and writing parts of the paper.

## Conflict of Interest Statement

The authors declare that the research was conducted in the absence of any commercial or financial relationships that could be construed as a potential conflict of interest.

## References

[B1] Aiken L. S. West S. G. (1991). *Multiple Regression: Testing and Interpreting Interactions.* Newbury Park, CA: Sage Publications.

[B2] Bandura A. (1982). Self-efficacy mechanism in human agency. *Am. Psychol.* 37 122–147. 10.1037/0003-066X.37.2.122

[B3] Barrick M. R. Stewart G. L. Neubert M. J. Mount M. K. (1998). Relating member ability and personality to work-team processes and team effectiveness. *J. Appl. Psychol.* 83 377–391. 10.1037/0021-9010.83.3.377

[B4] Baumeister R. F. Heatherton T. F. (1996). Self-regulation failure: an overview. *Psychol. Inq.* 7 1–15. 10.1207/s15327965pli0701_1

[B5] Bliese P. D. (2000). “Within-group agreement, non-independence, and reliability: implications for data aggregation and analysis,” in *Multilevel Theory, Research, and Methods in Organizations*, eds Klein K. J. Kozlowski S. W. J. (San Francisco, CA: Jossey-Bass), 349–381.

[B6] Bryk A. S. Raudenbush S. W. (1982). *Hierarchical Linear Models.* Thousand Oaks, CA: Sage.

[B7] Bunderson J. S. Sutcliffe K. M. (2003). Management team learning orientation and business unit performance. *J. Appl. Psychol.* 88 552–560. 10.1037/0021-9010.88.3.552 12814303

[B8] Button S. B. Mathieu J. E. Zajac D. M. (1996). Goal orientation in organizational research: a conceptual and empirical foundation. *Organ. Behav. Hum. Decis. Process.* 67 26–48. 10.1006/obhd.1996.0063

[B9] Campion M. A. Medsker G. J. Higgs A. C. (1993). Relations between work group characteristics and effectiveness: implications for designing effective work groups. *Pers. Psychol.* 46 823–850. 10.1111/j.1744-6570.1993.tb01571.x

[B10] Carver C. S. Scheier M. F. (1982). Control theory: a useful conceptual framework for personality-social, clinical, and health psychology. *Psychol. Bull.* 92 111–135. 10.1037/0033-2909.92.1.111 7134324

[B11] Chan D. (1998). Functional relations among constructs in the same content domain at different levels of analysis: a typology of composition models. *J. Appl. Psychol.* 83 234–246. 10.1037/0021-9010.83.2.234

[B12] Chang A. Bordia P. Duck J. (2003). Punctuated equilibrium and linear progression: toward a new understanding of group development. *Acad. Manage. J.* 46 106–117. 10.2307/30040680

[B13] Chen G. Gogus C. I. (2008). “Motivation in an of work teams: a multilevel perspective,” in *Work Motivation: Past, Present, and Future*, eds Kanfer R. Chen G. Pritchard R. D. (New York, NY: Routledge).

[B14] Chen G. Thomas B. Wallace J. C. (2005). A multilevel examination of the relationships among training outcomes, mediating regulatory processes, and adaptive performance. *J. Appl. Psychol.* 90 827–841. 10.1037/0021-9010.90.5.827 16162057

[B15] Chen G. C. Kanfer R. (2006). Toward a systems theory of motivated behavior in work teams. *Res. Organ. Behav.* 27 223–267. 10.1016/S0191-3085(06)27006-0

[B16] Corkin D. M. Yu S. L. Lindt S. F. (2011). Comparing active delay and procrastination from a self-regulated learning perspective. *Learn. Individ. Differ.* 21 602–606. 10.1016/j.lindif.2011.07.005

[B17] DeShon R. P. Kozlowski S. W. J. Schmidt A. M. Milner K. R. Wiechmann D. (2004). A multiple-goal, multilevel model of feedback effects on the regulation of individual and team performance. *J. Appl. Psychol.* 89 1035–1056. 10.1037/0021-9010.89.6.1035 15584840

[B18] Dragoni L. (2005). Understanding the emergence of state goal orientation in organizational work groups: the role of leadership and multilevel climate perceptions. *J. Appl. Psychol.* 90 1084–1095. 10.1037/0021-9010.90.6.1084 16316267

[B19] Dragoni L. Kuenzi M. (2012). Better understanding work unit goal orientation: its emergence and impact under different types of work unit structure. *J. Appl. Psychol.* 97 1032–1048. 10.1037/a0028405 22545620

[B20] Dweck C. S. (1986). Motivational processes affecting learning. *Am. Psychol.* 41 1040–1048. 10.1037/0003-066X.41.10.1040

[B21] Dweck C. S. Leggett E. L. (1988). A social-cognitive approach to motivation and personality. *Psychol. Rev.* 95 256–273. 10.1037/0033-295X.95.2.256

[B22] Elliot A. J. (1999). Approach and avoidance motivation and achievement goals. *Educ. Psychol.* 34 169–189. 10.1207/s15326985ep3403_3

[B23] Elliot A. J. Harackiewicz J. M. (1996). Approach and avoidance achievement goals and intrinsic motivation: a mediational analysis. *J. Pers. Soc. Psychol.* 70 461–475. 10.1037/0022-3514.70.3.461 8014838

[B24] Elliot A. J. McGregor H. A. (2001). A 2 × 2 achievement goal framework. *J. Pers. Soc. Psychol.* 80 501–519. 10.1037/0022-3514.80.3.501 11300582

[B25] Ferrari J. R. (1992). Procrastination in the workplace: attributions for failure among individuals with similar behavioral tendencies. *Pers. Individ. Dif.* 13 315–319. 10.1016/0191-8869(92)90108-2

[B26] Ferrari J. R. (1993). Christmas and procrastination: explaining lack of diligence at a “real-world” task deadline. *Pers. Individ. Dif.* 14 25–33. 10.1016/0191-8869(93)90171-X

[B27] Ferrari J. R. Diaz-Morales J. F. O’Callaghan J. Argumedo K. D. D. (2007). Frequent behavioral delay tendencies by adults: international prevalence rates of chronic procrastination. *J. Cross Cult. Psychol.* 38 458–464. 10.1177/0022022107302314

[B28] Ferrari J. R. Johnson J. L. McCown W. G. (1995). *Procrastination and Task Avoidance: Theory, Research, and Treatment.* New York, NY: Plenum 10.1007/978-1-4899-0227-6

[B29] Ferrari J. R. Patel T. (2004). Social comparisons by procrastinators: rating peers with similar or dissimilar delay tendencies. *Pers. Individ. Dif.* 37 1493–1501. 10.1016/j.paid.2004.02.006

[B30] Ferrari J. R. Tice D. M. (2000). Procrastination as a self-handicap for men and women: a task-avoidance strategy in a laboratory setting. *J. Res. Pers.* 34 73–83. 10.1006/jrpe.1999.2261

[B31] Gersick C. J. G. (1988). Time and transition in work teams: toward a new model of group development. *Acad. Manage. J.* 31 9–41. 10.2307/256496

[B32] Gersick C. J. G. (1989). Marking time: predictable transitions in task groups. *Acad. Manage. J.* 32 274–309. 10.2307/256363

[B33] Gevers J. M. P. Van Eerde W. Rutte C. G. (2009). Team self-regulation and meeting deadlines in project teams: antecedents and effects of temporal consensus. *Eur. J. Work Organ. Psychol.* 18 295–321. 10.1080/13594320701693217

[B34] Gully S. M. Incalcaterra K. A. Joshi A. Beaubien J. M. (2002). A meta-analysis of team-efficacy, potency, and performance: interdependence and level of analysis as moderators of observed relationships. *J. Appl. Psychol.* 87 819–832. 10.1037/0021-9010.87.5.819 12395807

[B35] Hansen R. S. (2006). Benefits and problems with student teams: suggestions for improving team projects. *J. Educ. Bus.* 82 11–19. 10.3200/JOEB.82.1.11-19

[B36] Harackiewicz J. M. Barron K. E. Pintrich P. R. Elliot A. J. Thrash T. M. (2002). Revision of achievement goal theory: necessary and illuminating. *J. Educ. Psychol.* 94 638–645. 10.1037/0022-0663.94.3.638

[B37] Harriott J. Ferrari J. R. (1996). Prevalence of procrastination among samples of adults. *Psychol. Rep.* 78 611–616. 10.2466/pr0.1996.78.2.611

[B38] Hayes A. F. (2013). *Introduction to Mediation, Moderation, and Conditional Process Analysis: A Regression-based Approach.* New York, NY: Guilford Press.

[B39] Hembree R. (1988). Correlates, causes, effects, and treatment of test anxiety. *Rev. Educ. Res.* 58 47–77. 10.3102/00346543058001047

[B40] Hofmann D. A. Jones L. M. (2005). Leadership, collective personality, and performance. *J. Appl. Psychol.* 90 509–522. 10.1037/0021-9010.90.3.509 15910146

[B41] Howell A. J. Buro K. (2009). Implicit beliefs, achievement goals, and procrastination: a mediational analysis. *Learn. Individ. Dif.* 19 151–154. 10.1016/j.lindif.2008.08.006

[B42] Howell A. J. Watson D. C. (2007). Procrastination: associations with achievement goal orientation and learning strategies. *Pers. Individ. Dif.* 43 167–178. 10.1016/j.paid.2006.11.017

[B43] Ilgen D. R. (1999). Teams embedded in organizations. *Am. Psychol.* 54 129–139. 10.1037/0003-066X.54.2.129

[B44] Ilgen D. R. Hollenbeck J. R. Johnson M. Jundt D. (2005). Teams in organizations: from input-process-output models to IMOI models. *Annu. Rev. Psychol.* 56 517–543. 10.1146/annurev.psych.56.091103.070250 15709945

[B45] Inzlicht M. Legault L. Teper R. (2014). Exploring the mechanisms of self-control improvement. *Curr. Dir. Psychol. Sci.* 23 302–307. 10.1177/0963721414534256

[B46] Karau S. J. Williams K. D. (1993). Social loafing: a meta-analytic review and theoretical integration. *J. Pers. Soc. Psychol.* 65 681–706. 10.1037/0022-3514.65.4.681

[B47] Klein K. J. Conn A. B. Smith D. B. Sorra J. S. (2001). Is everyone in agreement? An exploration of within-group agreement in employee perceptions of the work environment. *J. Appl. Psychol.* 86 3–16. 10.1037/0021-9010.86.1.3 11302231

[B48] Klein K. J. Kozlowski S. W. J. (2000). From micro to meso: critical steps in conceptualizing and conducting multilevel research. *Organ. Res. Methods* 3 211–236. 10.1177/109442810033001

[B49] Kozlowski S. W. J. Ilgen D. R. (2006). Enhancing the effectiveness of work groups and teams. *Psychol. Sci. Public Interest* 7 77–124. 10.1111/j.1529-1006.2006.00030.x 26158912

[B50] Kristof-Brown A. L. Zimmerman R. D. Johnson E. C. (2005). Consequences of individuals’ fit at work: a meta-analysis of person-job, person-organization, person-group, and person-supervisor fit. *Pers. Psychol.* 58 281–342. 10.1111/j.1744-6570.2005.00672.x

[B51] Lay C. H. (1986). At last, my research article on procrastination. *J. Res. Pers.* 20 474–495. 10.1016/0092-6566(86)90127-3

[B52] LeBreton J. M. Senter J. L. (2008). Answers to 20 questions about interrater reliability and interrater agreement. *Organ. Res. Methods* 11 815–852. 10.1177/1094428106296642

[B53] Lindell M. K. Brandt C. J. Whitney D. J. (1999). A revised index of interrater agreement for multi-item ratings of a single target. *Appl. Psychol. Meas.* 23 127–135. 10.1177/01466219922031257

[B54] Linnenbrink E. A. (2005). The dilemma of performance-approach goals: the use of multiple goal contexts to promote students’ motivation and learning. *J. Educ. Psychol.* 97 197–213. 10.1037/0022-0663.97.2.197

[B55] Lovibond P. F. Lovibond S. H. (1995). The structure of negative emotional states: comparison of the depression anxiety stress scales (DASS) with the Beck depression and anxiety inventories. *Behav. Res. Ther.* 33 335–343. 10.1016/0005-7967(94)00075-U 7726811

[B56] Maltarich M. A. Greenwald J. Reilly G. (2016). Team-level goal orientation: an emergent state and its relationships with team inputs, process, and outcomes. *Eur. J. Work Organ. Psychol.* 25 68–88. 10.1080/1359432X.2015.1004318

[B57] Marks M. A. Mathieu J. E. Zaccaro S. J. (2001). A temporally based framework and taxonomy of team processes. *Acad. Manage. Rev.* 26 356–376.

[B58] Marks M. A. Panzer F. J. (2004). The influence of team monitoring on team processes and performance. *Hum. Perform.* 17 25–41. 10.1207/S15327043HUP1701_2

[B59] Mathieu J. Maynard M. T. Rapp T. Gilson L. (2008). Team effectiveness 1997-2007: a review of recent advancements and a glimpse into the future. *J. Manage.* 34 410–476. 10.1177/0149206308316061

[B60] Mathieu J. E. Tannenbaum S. I. Donsbach J. S. Alliger G. M. (2014). A review and integration of team composition models: moving toward a dynamic and temporal framework. *J. Manage.* 40 130–160. 10.1177/0149206313503014

[B61] Mehta A. Feild H. S. Armenakis A. Mehta N. (2009). Team goal orientation and team performance: the mediating role of team planning. *J. Manage.* 35 1026–1046. 10.1177/0149206308326773

[B62] Milgram N. Toubiana Y. (1999). Academic anxiety, academic procrastination, and parental involvement in students and their parents. *Br. J. Educ. Psychol.* 69 345–361. 10.1348/000709999157761 10549240

[B63] Nguyen B. Steel P. Ferrari J. R. (2013). Procrastination’s impact in the workplace and the workplace’s impact on procrastination. *Int. J. Sel. Assess.* 21 388–399. 10.1111/ijsa.12048

[B64] Noordzij G. Van Hooft E. A. J. Van Mierlo H. Van Dam A. Born M. P. (2013). The effects of a learning-goal orientation training on self-regulation: a field experiment among unemployed job seekers. *Pers. Psychol.* 66 723–755. 10.1111/peps.12011

[B65] Payne S. C. Youngcourt S. S. Beaubien J. M. (2007). A meta-analytic examination of the goal orientation nomological net. *J. Appl. Psychol.* 92 128–150. 10.1037/0021-9010.92.1.128 17227156

[B66] Podsakoff P. M. MacKenzie S. B. Lee J.-Y. Podsakoff N. P. (2003). Common method biases in behavioral research: a critical review of the literature and recommended remedies. *J. Appl. Psychol.* 88 879–903. 10.1037/0021-9010.88.5.879 14516251

[B67] Porter C. O. Webb J. W. Gogus C. I. (2010). When goal orientations collide: effects of learning and performance orientation on team adaptability in response to workload imbalance. *J. Appl. Psychol.* 95 935–943. 10.1037/a0019637 20718514

[B68] Rapp T. L. Bachrach D. G. Rapp A. A. Mullins R. (2014). The role of team goal monitoring in the curvilinear relationship between team efficacy and team performance. *J. Appl. Psychol.* 99 976–987. 10.1037/a0036978 24865579

[B69] Rawsthorne L. J. Elliot A. J. (1999). Achievement goals and intrinsic motivation: a meta-analytic review. *Pers. Soc. Psychol. Rev.* 3 326–344. 10.1207/s15327957pspr0304_3 15661680

[B70] Riggs M. L. Knight P. A. (1994). The impact of perceived group success-failure on motivational beliefs and attitudes: a causal model. *J. Appl. Psychol.* 79 755–766. 10.1037/0021-9010.79.5.755 7989276

[B71] Salas E. Dickinson T. L. Converse S. A. Tannenbaum S. I. (1992). “Toward an understanding of team performance,” in *Teams, their Training and Performance*, eds Swezey R. W. Salas E. (Norwood, NJ: Ablex Publishing).

[B72] Scher S. J. Osterman N. M. (2002). Procrastination, conscientiousness, anxiety, and goals: exploring the measurement and correlates of procrastination among school-aged children. *Psychol. Sch.* 39 385–398. 10.1002/pits.10045

[B73] Scullen S. E. Mount M. K. Goff M. (2000). Understanding the latent structure of job performance ratings. *J. Appl. Psychol.* 85 956–970. 10.1037/0021-9010.85.6.956 11125659

[B74] Seo E. H. (2009). The relationship of procrastination with a mastery goal versus an avoidance goal. *Soc. Behav. Pers.* 37 911–920. 10.2224/sbp.2009.37.7.911

[B75] Seo M. Ilies R. (2009). The role of self-efficacy, goal, and affect in dynamic motivational self-regulation. *Organ. Behav. Hum. Decis. Process.* 109 120–133. 10.1016/j.obhdp.2009.03.001

[B76] Sirois F. M. (2014). Out of sight, out of time? A meta-analytic investigation of procrastination and time perspective. *Eur. J. Pers.* 28 511–520. 10.1002/per.1947

[B77] Sirois F. M. Melia-Gordon M. L. Pychyl T. A. (2003). “I’ll look after my health, later”: an investigation of procrastination and health. *Pers. Individ. Dif.* 35 1167–1184. 10.1016/S0191-8869(02)00326-4

[B78] Solomon L. J. Rothblum E. D. (1984). Academic procrastination: frequency and cognitive-behavioral correlates. *J. Couns. Psychol.* 91 503–509. 10.1037/0022-0167.31.4.503

[B79] Stajkovic A. D. Lee D. Nyberg A. J. (2009). Collective efficacy, group potency, and group performance: meta-analyses of their relationships, and test of a mediation model. *J. Appl. Psychol.* 94 814–828. 10.1037/a0015659 19450017

[B80] Stajkovic A. D. Luthans F. (1998). Self-efficacy and work related performance. A meta-analysis. *Psychol. Bull.* 124 240–261. 10.1037/0033-2909.124.2.240

[B81] Steel P. (2007). The nature of procrastination: a meta-analytic and theoretical review of quintessential self-regulatory failure. *Psychol. Bull.* 133 65–94. 10.1037/0033-2909.133.1.65 17201571

[B82] Steel P. (2010). Arousal, avoidant and decisional procrastinators: Do they exist? *Pers. Individ. Dif.* 48 926–934. 10.1016/j.paid.2010.02.025

[B83] Steel P. König C. J. (2006). Integrating theories of motivation. *Acad. Manage. Rev.* 31 889–913. 10.5465/AMR.2006.22527462

[B84] Svartdal F. Steel P. (2017). Irrational delay revisited: examining five procrastination scales in a global sample. *Front. Psychol.* 8:1927. 10.3389/fpsyg.2017.01927 29163302 PMC5676095

[B85] Tice D. M. Baumeister R. F. (1997). Longitudinal study of procrastination, performance, stress, and health: the costs and benefits of dawdling. *Psychol. Sci.* 8 454–458. 10.1111/j.1467-9280.1997.tb00460.x

[B86] Tice D. M. Bratslavsky E. Baumeister R. F. (2001). Emotional distress regulation takes precedence over impulse control: if you feel bad, do it! *J. Pers. Soc. Psychol.* 80 53–67. 10.1037/0022-3514.80.1.53 11195891

[B87] Urdan T. (2004). Predictors of academic self-handicapping and achievement: examining achievement goals, classroom goal structures, and culture. *J. Educ. Psychol.* 96 251–264. 10.1037/0022-0663.96.2.251

[B88] Van Dyck C. Van Hooft E. A. J. De Gilder D. Liesveld L. C. (2010). Proximal antecedents and correlates of adopted error approach: a self-regulatory perspective. *J. Soc. Psychol.* 150 428–451. 10.1080/00224540903366743 21058573

[B89] Van Eerde W. (2000). Procrastination: self-regulation in initiating aversive goals. *Appl. Psychol. Int. Rev.* 49 372–389. 10.1111/1464-0597.00021

[B90] Van Eerde W. (2003). A meta-analytically derived nomological network of procrastination. *Pers. Individ. Dif.* 35 1401–1418. 10.1016/S0191-8869(02)00358-6

[B91] Van Hooft E. A. J. (2010). “The role of task-related factors in predicting procrastinatory behavior,” in *Proceedings of the 25th Annual Meeting of the Society of Industrial and Organizational Psychology: Why do We Put Things Off? Self-regulation, Task-characteristics, and Procrastination*, Atlanta, GA.

[B92] Van Mierlo H. Kleingeld A. (2010). Goals, strategies, and group performance: some limits of goal setting in groups. *Small Group Res.* 41 524–555. 10.1177/1046496410373628

[B93] Van Mierlo H. Van Hooft E. A. J. (2015). A group-level conceptualization of the 2x2 achievement goal framework: antecedents and motivational outcomes. *Group Organ. Manage.* 40 776–808. 10.1177/1059601115592990

[B94] Van Mierlo H. Vermunt J. K. Rutte C. G. (2009). Composing group-level constructs from individual-level survey data. *Organ. Res. Methods* 12 368–392. 10.1177/1094428107309322

[B95] Van Yperen N. W. (2006). A novel approach to assessing achievement goals in the context of the 2 x 2 framework: identifying distinct profiles of individuals with different dominant achievement goals. *Pers. Soc. Psychol. Bull.* 32 1432–1445. 10.1177/0146167206292093 17030886

[B96] Vancouver J. B. Kendall L. (2006). When self-efficacy negatively relates to motivation and performance in a learning context. *J. Appl. Psychol.* 91 1146–1153. 10.1037/0021-9010.91.5.1146 16953775

[B97] Vancouver J. B. More K. M. Yoder R. J. (2008). Self-efficacy and resource allocation: support for a nonmonotonic, discontinuous model. *J. Appl. Psychol.* 93 35–47. 10.1037/0021-9010.93.1.35 18211133

[B98] Vandewalle D. (1997). Development and validation of a work domain goal orientation instrument. *Educ. Psychol. Meas.* 57 995–1015. 10.1177/0013164497057006009

[B99] Williams H. M. Meân L. J. (2004). Measuring gender composition in work groups: a comparison of existing methods. *Organ. Res. Methods* 7 456–474. 10.1177/1094428104269175

[B100] Williams K. D. (2010). Dyads can be groups (and often are). *Small Group Res.* 41 268–274. 10.1177/1046496409358619

[B101] Wolters C. A. (2003). Understanding procrastination from a self-regulated learning perspective. *J. Educ. Psychol.* 95 179–187. 10.1037/0022-0663.95.1.179

[B102] Wolters C. A. (2004). Advancing achievement goal theory: using goal structures and goal orientations to predict students’ motivation, cognition, and achievement. *J. Educ. Psychol.* 96 236–250. 10.1037/0022-0663.96.2.236

